# Identification of validated case definitions for chronic disease using electronic medical records: a systematic review protocol

**DOI:** 10.1186/s13643-017-0431-9

**Published:** 2017-02-23

**Authors:** Sepideh Souri, Nicola E. Symonds, Azin Rouhi, Brendan C. Lethebe, Stephanie Garies, Paul E. Ronksley, Tyler S. Williamson, Gabriel E. Fabreau, Richard Birtwhistle, Hude Quan, Kerry A. McBrien

**Affiliations:** 1 Department of Community Health Sciences, Cumming School of Medicine, University of Calgary, Calgary, Alberta Canada; 2 Faculty of Science, University of British Columbia, Vancouver, Canada; 3 Faculty of Medicine and Dentistry, University of Alberta, Edmonton, Canada; 4 Department of Family Medicine, Cumming School of Medicine, University of Calgary, Calgary, Alberta Canada; 5 Department of Medicine, Cumming School of Medicine, University of Calgary, Calgary, Alberta Canada; 6 Department of Family Medicine, Faculty of Health Sciences, Queen’s University, Kingston, Ontario Canada

**Keywords:** Systematic review, Electronic medical record, Chronic disease, Case definitions, Big data

## Abstract

**Background:**

Primary care electronic medical record (EMR) data are being used for research, surveillance, and clinical monitoring. To broaden the reach and usability of EMR data, case definitions must be specified to identify and characterize important chronic conditions. The purpose of this study is to identify all case definitions for a set of chronic conditions that have been tested and validated in primary care EMR and EMR-linked data. This work will provide a reference list of case definitions, together with their performance metrics, and will identify gaps where new case definitions are needed.

**Methods:**

We will consider a set of 40 chronic conditions, previously identified as potentially important for surveillance in a review of multimorbidity measures. We will perform a systematic search of the published literature to identify studies that describe case definitions for clinical conditions in EMR data and report the performance of these definitions. We will stratify our search by studies that use EMR data alone and those that use EMR-linked data. We will compare the performance of different definitions for the same conditions and explore the influence of data source, jurisdiction, and patient population.

**Discussion:**

EMR data from primary care providers can be compiled and used for benefit by the healthcare system. Not only does this work have the potential to further develop disease surveillance and health knowledge, EMR surveillance systems can provide rapid feedback to participating physicians regarding their patients. Existing case definitions will serve as a starting point for the development and validation of new case definitions and will enable better surveillance, research, and practice feedback based on detailed clinical EMR data.

**Systematic review registration:**

PROSPERO CRD42016040020

**Electronic supplementary material:**

The online version of this article (doi:10.1186/s13643-017-0431-9) contains supplementary material, which is available to authorized users.

## Background

### Rationale

The collection and storage of vast amounts of health data is growing rapidly [1]. These “big data” include electronic medical record (EMR) data and traditional coded administrative health data. EMRs, which contain comprehensive demographic and clinical information including diagnoses, prescriptions, physical measurements, and laboratory test results, are increasingly used in the primary care setting to record patient information and provide patient care [2]. EMR data are used for research, surveillance, and clinical monitoring in many countries; however, their potential is largely unused in Canada [3].

Administrative health data are routinely used for research and surveillance, as most are population-based, relatively inexpensive compared to primary data collection, and exist in a structured format [4]. Like administrative data, information contained in EMRs also has the potential to be collected in databases and used in research and public health surveillance [3]. EMR data can be used alone or in some cases linked to traditional coded administrative health data (EMR-linked data). An important step in conducting research using EMR data is to identify subgroups of patients with a specific disease or condition of interest using validated disease case definitions.

Case definitions, also referred to as phenotypes, are automated computerized algorithms applied to secondary data that allow for identification of specific cohorts within EMR databases without the need for manual chart review by a researcher or clinician [5]. In general, case definitions are validated against a gold standard for disease identification, most often manual review of patient charts. Researchers around the world have developed and validated case definitions for different disease conditions and applied them to EMR data. Validated disease case definitions have the potential to be modified and applied to various EMR databases to enable better surveillance, research, and practice feedback based on detailed clinical EMR data.

Chronic diseases are a significant burden to patients and the health care system. They include both physical and mental illnesses and affect at least one third of all Canadians [6]. Barnett et al. conducted a literature review, followed by a consensus exercise to identify a set of 40 conditions likely to be chronic and have significant impact on patients’ treatment needs, function, quality of life, morbidity, and mortality [7]. A systematic review of case definitions applied to administrative health data identified validated algorithms to detect 30 of these conditions [8]. No previous work has identified and reported on validated disease case definitions for chronic disease in EMR or EMR-linked data.

### Objective

The objective of this study is to identify all case definitions for a set of chronic conditions, which have been tested and validated in primary care EMR and EMR-linked data. We will conduct a systematic review of primary studies that report on the development and validation of chronic disease case definitions for use in primary care EMR and EMR-linked data. This work will allow us to collect and report on a comprehensive set of chronic conditions with validated case definitions. Not only will this be a valuable resource for researchers using EMR databases, but knowledge of these existing definitions will also pave the way for development and validation of additional case definitions for diseases where such definitions are lacking.

## Methods

We will perform a systematic review following a predetermined protocol, in accordance with the Preferred Reporting Items for Systematic Reviews and Meta-analyses (PRISMA) reporting guidelines [9].

### Data sources and search strategy

We will search MEDLINE and MEDLINE-in-Process (Ovid) and Embase (Ovid) with no date, country, or language restrictions. We will also search the bibliographies of all identified studies. Further, the websites for EMR and administrative databases will be searched for bibliographic lists (e.g., Clinical Practice Research Datalink [10], www.cprd.com), and content experts will be contacted for information about other potential ongoing or unpublished studies. The search of online databases will include three themes:

1. *Electronic medical records*
2. *Case definition*
3. *Validation study*

We will use a comprehensive set of MeSH terms and keyword searches for each of the three themes to ensure we capture all relevant references. For example, the term “EMR” may be synonymous with a number of relevant keywords (e.g., computerized medical records, electronic health record, EHR). These three searches will then be combined using the Boolean term “AND.” Additional file 1 outlines our detailed MEDLINE search strategy. Terms used to define chronic conditions will be intentionally omitted to ensure capture of any chronic condition, including our pre-specified list of 40 conditions as shown in Table  1 [7].

**Table 1:** List of the 40 chronic disease conditions (Barnett et al. [7])

• Hypertension • Depression • Painful condition • Asthma • Coronary heart disease • Treated dyspepsia • Diabetes • Thyroid disorders • Rheumatoid arthritis • Hearing loss • Chronic obstructive pulmonary disease • Anxiety and other somatoform disorders • Irritable bowel syndrome	• New diagnosis of cancer in last 5 years • Alcohol problems • Other psychoactive substance misuse • Treated constipation • Stoke and transient ischemic attack • Chronic kidney disease • Diverticular disease of the intestine • Atrial fibrillation • Peripheral vascular disease • Heart failure • Prostate disorders • Glaucoma	• Epilepsy • Dementia • Schizophrenia • Psoriasis or eczema • Inflammatory bowel disease • Migraine • Blindness and low vision • Chronic sinusitis • Learning disability • Anorexia or bulimia • Bronchiectasis • Parkinson’s disease • Multiple sclerosis • Viral hepatitis • Chronic liver disease

### Study selection

Two reviewers will independently screen all abstracts. Articles that report original data for the development and validation of chronic disease case definitions in primary care EMR data or EMR-linked data will be considered for further review. The initial screen will be intentionally broad to capture any relevant literature. All citations where either reviewer feels that further review is warranted will be kept for full text review. Agreement will be quantified at this stage using the kappa statistic, and any disagreements will be resolved by consensus or by a third reviewer as needed. Bibliographic details from all stages of the review will be managed with the *Synthesis* software package [11].

The same two reviewers will scan full text articles for the following inclusion criteria:

1. The database under study is either a primary care EMR database or a primary care EMR database linked to at least one administrative health database.
2. There is a description of a computerized case definition for a specific disease or condition.
3. The condition or conditions under study include at least one of the 40 chronic conditions identified by Barnett et al. [7].
4. A clearly stated reference standard is used to validate the case definition.
5. Validity outcomes are reported (i.e., sensitivity, specificity, positive predictive value, negative predictive value, kappa, receiver operating characteristic, likelihood ratio).

Exclusion criteria: Non-human studies will be excluded. The study will be limited to diseases that present in a primary care setting. Studies reporting on dental health or other non-primary care settings will be excluded. We will also exclude studies where EMR data is based on patient self-report.

### Data extraction

A data extraction form will be used to collect information from each included study. In duplicate, the following data elements will be extracted: publication date, first author, country, EMR platform, administrative data sources (in the case of linked studies), description of case definition, disease(s) under study, and measures of validity (e.g., sensitivity, specificity).

### Risk of bias assessment

Included studies will be assessed for quality using a component approach. We will use relevant items from the QUADAS quality assessment tool for diagnostic accuracy studies [12]. This tool includes an assessment of bias in several domains, including patient selection, the validation strategy, and reporting of outcomes. Two authors will independently assess risk of bias in each domain and report the risk of bias as high, low, or unclear. Disagreements will be resolved by discussion or with a third reviewer as needed.

### Data synthesis

The number of articles identified, including those that are included and excluded will be summarized using a flow chart. Results from included studies will be described in detail, grouped by disease or health condition, and reported for EMR and EMR-linked data separately. For each chronic condition, relevant elements from each study will be reported and summarized. Data will not be pooled, since there are several disease conditions and we anticipate finding heterogeneity between databases used across the different studies. We will stratify our findings by data source (number and type), jurisdiction, and patient population. Finally, given the complementary nature of our review with that done by Tonelli et al. on case definitions in administrative health data [8], we will produce a comparison table that describes case definitions and their metrics for each of the three major types of data: EMR data alone, EMR-linked data, and administrative data alone.

In addition to summarizing case definitions and their performance metrics by disease condition, we will also perform a secondary analysis focused on the methods employed across case definitions. We will produce a detailed inventory of the combinations of variables used, the data fields accessed, and the computer programming methods used. Within disease conditions for which there is more than one validated case definition, we will perform a descriptive analysis that compares the specifications of the case definitions and their relative performance.

## Discussion

Data collected in primary care EMRs is becoming an important resource for conducting research and understanding disease patterns and prevalence. The recent and widespread uptake of EMRs in primary care has created a new source of detailed clinical information not found in administrative health data that has the potential to be used in research and surveillance [1, 3]. An essential step in the use of EMR data in research is applying validated disease case definitions to identify a group of patients with a condition under study.

We undertook this project to collect and report on all studies that have developed and validated disease case definitions using EMR data. Validated case definitions are important tools, since they can be adapted and applied to different EMR databases to conduct research. In addition, this study will allow us to understand the extent of disease conditions for which validated case definitions have been developed and encourage further research to develop and validate case definitions for other disease conditions, where such definitions do not exist.

Specifically, our results will improve our ability to analyze chronic diseases at the population level and, further, examine the effects of multimorbidity. The existence of validated case definitions for EMRs will also allow precise characterization of individual patients, enabling physicians to tailor practice guidelines according to individual risk profiles, as well as enhance clinical feedback to physicians and practices by making quality metrics more specific to their practice panel. Additionally, this review will enable researchers to access the detailed clinical information contained in EMR data. Finally, our results will improve standardization of definitions used for disease conditions and will ultimately improve comparison of surveillance metrics at the international level.

## Additional file

Additional file 1: Proposed search strategy for Ovid MEDLINE®. (DOCX 62 kb)

## Abbreviations

EHR: Electronic health record
EMR: Electronic medical record
PRISMA: Preferred Reporting Items for Systematic Reviews and Meta-analyses
QUADAS: Quality Assessment of Diagnostic Accuracy Studies
