# Supplementary material for: Identification of validated case definitions for chronic disease using electronic medical records: a systematic review protocol
Source: Syst Rev. 2017 Feb 23;6:38. doi: 10.1186/s13643-017-0431-9 (PMC5322672; doi:10.1186/s13643-017-0431-9)
Supplement: Additional file 1: — Proposed search strategy for Ovid MEDLINE®. (DOCX 62 kb) [file 13643_2017_431_MOESM1_ESM.docx]

**Additional File 1: Proposed search strategy for Ovid MEDLINE®**

1. electronic medical record*.mp.
2. electronic health record*.mp.
3. EMR.mp.
4. EHR.mp.
5. exp Electronic Health Records/
6. exp Medical Records Systems, Computerized/
7. computerized medical record*.mp.
8. 1 or 2 or 3 or 4 or 5 or 6 or 7
9. algorithm*.mp.
10. case*mp.
11. disease*.mp.
12. comorbid*.mp.
13. condition*.mp.
14. diagnos*.mp.
15. defin*.mp.
16. ident*.mp.
17. phenotyp*.mp.
18. 9 or 10 or 11 or 12 or 13 or 14 or 15 or 16 or 17
19. valid*.mp.
20. accuracy.mp.
21. sensitivity.mp.
22. specificity.mp.
23. exp “Sensitivity and Specificity”/
24. positive predictive value.mp.
25. negative predictive value.mp.
26. PPV.mp.
27. NPV.mp.
28. Sp.mp.
29. Sn.mp.
30. 19 or 20 or 21 or 22 or 23 or 24 or 25 or 26 or 27 or 28 or 29
31. 8 and 18 and 30
